# Innovative Approaches to Extracting Phenolics from *Echinacea purpurea*: Maximizing Yield and Efficacy

**DOI:** 10.3390/foods14132325

**Published:** 2025-06-30

**Authors:** Mateja Senica, Gregor Mlinšek, Denis Rusjan, Maja Mikulic-Petkovsek

**Affiliations:** 1 Chair for Fruit Growing, Viticulture and Vegetable Growing, Biotechnical Faculty, Department of Agronomy, University of Ljubljana, Jamnikarjeva 101, 1000 Ljubljana, Slovenia; matejavoncina@gmail.com (M.S.); denis.rusjan@bf.uni-lj.si (D.R.); 2 Center for Kidney Transplantation, Department of Nephrology, University Medicinal Center Ljubljana, Zaloška 7, 1000 Ljubljana, Slovenia; gregor.mlinsek@gmail.com

**Keywords:** purple coneflower, phenolic compounds, extraction efficiency, solvents, functional ingredients

## Abstract

People have long been interested in plants rich in secondary metabolites and have tried to isolate them outside the plants. Purple coneflower is a crucial medicinal plant, known for its broad spectrum of bioactive substances. The type of solvent and the duration of maceration had an important impact on the phenolic level of purple coneflower leaves, flowers and roots. The flowers and leaves had a significantly higher phenolic concentration than the roots. The results of this study show the importance of stabilizing and regulating the polarity of the solvent and the duration of maceration to obtain the highest yield of phenolics from purple coneflower. The greatest yield of phenolics was gained with two natural extraction solvents, 5% acetic acid and glycerol, after 3 days of maceration, yielding (1696.05 mg 100 g^−1^ DW) and (2796.94 mg 100 g^−1^ DW) from the flowers of purple coneflower, respectively. For purple coneflower leaves and roots, the best extraction method was 40% ethanol after 3 days of maceration, and the total content analyzed was 1022.43 mg 100 g^−1^ DW and 1011.32 mg 100 g^−1^ DW, respectively. Different phenolics respond significantly to different maceration factors, but the best product from the flowers of purple coneflower is glycerol extract after 9 days of maceration. From the leaves of purple coneflower, we obtained the highest phenolic yield when producing extract in glycerol, a 40% ethanol tincture, or an acetic acid product after 3 days of maceration.

## 1. Introduction

People are increasingly interested in plants that can be used as functional foods. Functional foods, commonly referred to as nutraceuticals, are broadly defined as processed foods that offer health or medical benefits, including a potential reduction in the risk of disease [1]. In particular, plants that have a high content of secondary metabolites, especially phenolic compounds, contribute to the functional properties of medicinal plants. For many centuries, attempts have been made to isolate such substances from plants. Over the years, several extraction methods have been developed to obtain ingredients with functional properties from various plant materials, especially herbs, fruits, and vegetables. The main aim of extraction is to isolate the soluble compounds from the plants and discard the insoluble cellular residues. It is imperative that the processes are simple, inexpensive, fast, and environmentally friendly and that the composition of the functional compounds is preserved during extraction [2,3,4].

Phenolic compounds are secondary metabolites widespread in the plant kingdom. They have been shown to exert significant benefits on human well-being, reducing the risk of various diseases, especially those triggered by oxidative stress [4,5]. These bioactive compounds, extracted among different plant parts, differ depending on factors such as solvent type, extraction time, solvent concentration, and particle size of samples [6,7].

Phenolic compounds can contain various hydroxyl groups that can form binds with acids, sugars, or alkyl groups. As a result, the polarity of these components varies significantly, making it challenging to establish a universal method for the effective extraction of most phenolics [8]. Maceration is a widely used extraction technique in natural product research in which the choice of solvent plays a decisive role in the efficiency and selectivity of the extracted compounds [9]. Polar solvents such as water, ethanol and their mixtures are frequently used as they can extract a broad spectrum of bioactive compounds [10,11]. The polarity, concentration and composition of the solvent have a significant influence on the solubility of the target compounds as well as on the extraction yield [12]. For example, hydroalcoholic mixtures (e.g., 70% ethanol) are often reported to provide a good balance between polarity and extraction efficiency, especially for phenolic compounds [13]. In addition, process parameters such as extraction time, temperature, pH, and solvent-to-solid ratio can further influence the extraction result [10,14]. Therefore, careful selection and optimization of these variables is essential to maximize the recovery of the desired phytochemicals [9,12,15,16,17].

*Echinacea purpurea*, commonly known as the purple coneflower, is one of the most widely utilized medicinal plants in Europe and North America. It belongs to the Asteraceae family. It is a widely known traditional medicinal herb, used to prevent and treat colds, flus and coughs [18,19]. Besides having some nitrogen containing compounds, essential oils, polysaccharides, and glycoproteins, each plant part also accumulates quite high levels of phenolics with health promoting properties [20,21]. All these compounds from different plant parts in synergism attribute to a pharmacological effect on human health [22]. Bioactive compounds from purple coneflower have been shown to have immune stimulative, antimicrobial, antiviral, anti-inflammatory, anti-cancer, and antioxidant properties [22,23,24]. The most extensively used products made from purple coneflower are teas, liquid extracts, and tablets [23].

Due to the growing interest of people and the pharmaceutical industry in the importance of functional foods for the prevention of various modern-day diseases and ailments, public interest in the possibility of using plants with health-promoting properties has increased. All organs of the purple coneflower can be utilized as a suitable source for use as functional foods due to their proven health effects. As there are no such studies carried out in purple coneflower or any other medicinal plant, we proposed this study as a great contribution to science and all herbalists, who nowadays prepare on their own different extracts from medicinal plants. We therefore investigated the transition and stability of phenolics in different extraction solvents and duration of maceration for purple coneflower flower, leaf, and root. In this study four different solvent types were involved, such as pure water (H_2_O), 40% ethanol (40% EtOH), glycerol (Gly), and 5% acetic acid (5% AA) and three different extraction times (3, 6, and 9 days). Previous studies have revealed important insight on the influence of extraction time and solvent among different purple coneflower plant parts. The essence of this study was to present the best extraction method, which carried out the maximum yield of bioactive constituents from purple coneflower that are useful in human health.

## 2. Materials and Methods

### 2.1. Plant Materials

Roots, flowers, and leaves of purple coneflower (*Echinacea purpurea* (L.) Moench) were sampled in the field at the Škofja Loka site (46°11′28.28″ N; 14°19′27.74″ E; 369.8 m altitude; Slovenia). Plant organs were cleaned and immediately prepared for extraction.

### 2.2. Chemicals

We purchased following standards from Sigma-Aldrich Chemie (Steinheim, Germany): chlorogenic, caftaric and chicoric acid, naringenin, quercetin-3-*O*-galactoside, and kuromanin, while isotrifoliin, rutin, *p*-coumaric acid, astragalin were obtained from Fluka Chemie (Buchs, Switzerland), and caffeic acid from Roth (Karlsruhe, Germany). Ethanol and acetic acid were used as extraction solvents, while acetonitrile and formic acid, used as components of the mobile phase in HPLC and MS systems, were bought from Sigma-Aldrich Chemie. Glycerol used as extraction solvent was acquired from Farmalabor (Farmacisti Associati, Canosa di Puglla, Italia). Double-distilled water obtained from the Milli-Q system (Millipore, Bedford, MA, USA) was utilized both as an extraction solvent and as a component of the mobile phase.

### 2.3. Extraction of Different Plant Organs from Purple Cornflower

In this study, each part of the purple coneflower (flowers, leaves, and roots) was macerated in four different solvents (H_2_O, 40% EtOH, glycerol and 5% acetic acid) for a duration of 3, 6, and 9 days. The extraction was performed six times for each treatment and carried out in 100 mL round bottom flasks. The solid/liquid ratio and solvent concentration used in the maceration trials were selected based on a combination of relevant literature, preliminary optimization trials and previous experience with similar plant materials. In particular, a ratio of 1:28 *w*/*v* (leaves, flowers) and 1:7.14 *w*/*v* for roots proved to be a good balance between extraction efficiency. To prepare extracts from the flowers and leaves of purple coneflower, 2.5 g of dried plant material was individually combined with 70 mL of each solvent (H_2_O, 40% ethanol, glycerol, and 5% acetic acid) for each treatment. For root extracts, 7 g of dried root material was treated with 50 mL of each solvent under the same experimental conditions. The percentage of dry weight of the individual plant parts was as follows: flowers 32.7%, roots 43.5%, and leaves 59.3%. The samples were afterwards macerated in open flasks at room temperature for a total of 9 days. The contents of phenolics were evaluated and quantified every 3 days during 9 days of maceration with the aid of high-performance liquid chromatography (HPLC) coupled with mass spectrophotometry (MS).

### 2.4. Determination of Phenolic Content

All individual phenolic components were identified using a mass spectrometer (MS) with an LCQ Deca XP MAX (Thermo Finigan, San Jose, CA, USA) instrument, employing electrospray ionization (ESI) in both positive and negative ionization modes. Separation of phenolics was achieved using a Gemini C18 column (Phenomenex, Torrance, CA, USA) with a gradient solvent system consisting of 0.1% formic acid, acetonitrile, and double-distilled water, as described by Mikulic-Petkovsek et al. [25].

Phenolic compounds were identified by fragmentation patterns, retention times, UV-Vis spectral data (200–550 nm) and by spiking the samples with standard solutions.

### 2.5. Statistical Analysis

Statistical analysis was conducted using the R Commander software (R Formation for Statistical Computing, Auckland, New Zealand). A one-way analysis of variance (ANOVA) was employed to evaluate the data. The differences between the contents of each treatment were assessed using the Least Significant Difference (LSD) test.

## 3. Results and Discussion

**Figure 1:**
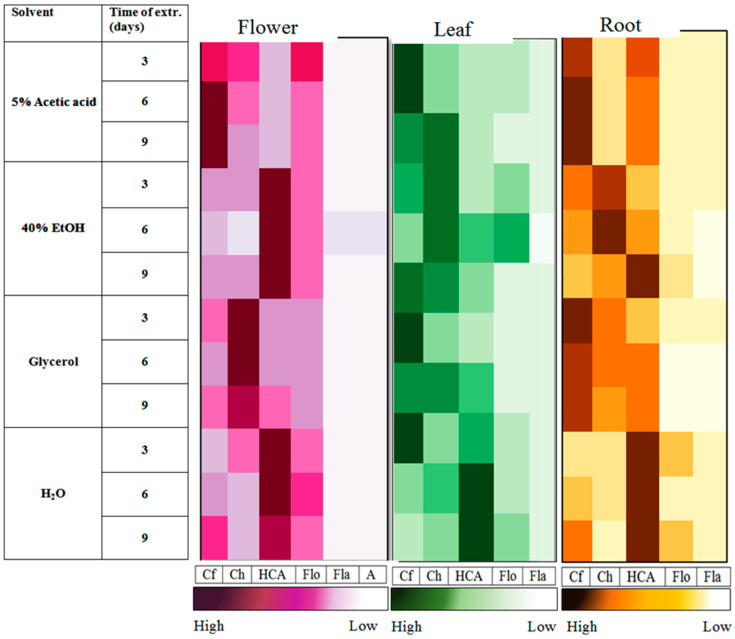
The contents of caftaric and chicoric acid and phenolic groups (HCA—hydroxycinnamic acids, Flo—flavonols, Fla—flavanones, A—anthocyanins) among different treatments for purple coneflower flower, leaf and root. Darker colour in the Figure means higher concentration of compound.

Among the different parts of purple coneflower, we identified twenty different phenolics in the flower, fourteen in the leaf and thirteen in the root (Table 1). In total, there were 24 different individual phenolic constituents (Table 1). The individual phenolics were classified into four groups: hydroxycinnamic acids, flavanones (naringenin derivatives), flavonols, and anthocyanins. Caftaric and chicoric acid are derivatives of caffeic acid, belonging to the group of hydroxycinnamic acids. Both exist in their *cis* and *trans* isomeric form. In Figure 1 and Table 2 were two major compounds separately presented in *trans* form, while *cis* isomers added to group of hydroxycinnamic acids. The highest peak in the chromatogram was chicoric acid, characterized by a pseudomolecular ion at *m*/*z* 473 and their fragmentation ions at *m*/*z* 311 [MS^2^] and *m*/*z* 179, 149, 293, and 131 [MS^3^] (see Table 1). The second most abundant peak in purple coneflower corresponded to caftaric acid with one molecular ion at *m*/*z* 311 and three [MS^2^] fragment ions at *m*/*z* 179, 149, and 135 (Table 1). The *trans* isomers of chicoric and caftaric acid accounted for about 30% to 50% of the phenolic contents analyzed in this study. That is in agreement with Wu et al. [26] and Lekar et al. [2]’s study, who found caffeic acid derivatives as major phenolic components in the *Echinacea purpurea* extracts. Both phenolic acids, chicoric, and caftaric acids are associated with remarkable health benefits due to their antioxidant, anti-inflammatory, and immunomodulatory properties [27,28] and chicoric acid also help regulate blood sugar [29]. Caftaric acid has shown potential in reducing oxidative stress, modulating inflammatory pathways and even protecting against neurodegenerative damage [30].

Phenolic contents statistically differed significantly among the different plant parts (flower, leaf, or root) and by type of solvent used (Table 2), with highly significant differences observed (*p* < 0.001; ***) across all individual groups of phenolics. Time of maceration, an additional factor in our study, also influenced the contents, but to a lower extent (*p* ≤ 0.05; *, NS) with the exception of caftaric acid, where was a strong statistical difference (***), which is in agreement with the results of Liaudaskas et al. [31]. Accordingly, the interaction between plant parts and time of maceration were not statistically significant for all phenolics (the exception was group of flavanones), while time of maceration combined with different solvents did not bring differences in the flavonol group (Table 2). In general, all three studied treatments (plant part, solvent, time of maceration) significantly influenced statistically the contents of individual as total phenolics (Table 2).

### 3.1. Comparison of Phenolic Contents Among Different Purple Coneflower Plant Part

**Figure 2:**
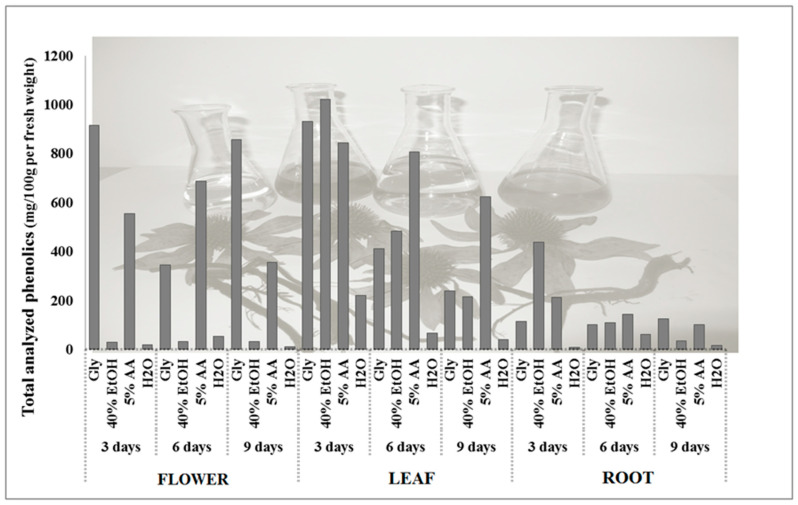
The contents of total analyzed phenolics among different treatments for purple coneflower flower, leaf, and root in mg per 100 g of fresh weight.

Significant variations in phenolic compound levels were observed among the different parts of the plant [7,23]. Our results revealed that, among the different plant parts, the flowers contained the highest total phenolic content (2796.94 mg per 100 g dry weight), followed by the leaves (1022.43 mg per 100 g dry weight), and the roots (1011.32 mg per 100 g dry weight) (Table 2). Flowers had the highest values among all treatments in the chicoric and caftaric acid, hydroxycinnamic acid, flavonols, and anthocyanin group as sum of total analyzed phenolics; the exception was total flavanones, which had the highest values in purple coneflower leaves. The flowers consisted of a higher proportion of water than the leaves and roots; accordingly, the contents of the total phenolics analyzed differed according to fresh weight. The leaves of the purple coneflower were in first place, followed by the flowers, and in last place, with the lowest content of phenolics, the roots (Figure 2). Accordingly, the highest total phenolic content from fresh purple coneflower products can be obtained from the leaves macerated in 40% ethanol, glycerol, or 5% acetic acid after 3 days of maceration (Figure 2). When we prepared the purple coneflower product from fresh or dried flowers, we used glycerol as a solvent in which the flowers were macerated for 3 or 9 days (Table 2; Figure 2).

Caftaric and chicoric acid, predominant phenolic acids in all purple coneflower parts (Table 1), presented from 20 to 60% of total phenolic contents for all purple coneflower parts. After the two major phenolic acids, the group of hydroxycinnamic acids followed with 10–20% in the flowers and 20–40% in the leaves and roots. The group of flavonols contributed less than 20% and the flavanones less than 3% to the total analyzed phenolic (TAP) content. The highest share of flavanones was analyzed in purple coneflower roots. Anthocyanins, presented only in the flowers, contributed only 1% of TAP in flower (Supplementary Figure S1).

There are a variety of reasons that influence the proportion of phenolics in various plant parts. The primary function of phenolics in plants is connected to the plant’s adaptation to specific environmental factors [32,33]. Therefore, the leaves and flowers of the purple coneflower are above ground and thus more exposed to environmental stresses such as participation, duration of snow cover, wind speed and intensity of ultraviolet radiation [32,34], pathogens, insect infestation, and injuries [5,35]. As a result, the accumulation of protective substances changes with the growing season and in various plant organs (vacuoles and cell nuclei) [36]. All the factors described above are consistent with the results of this study, which showed higher phenolic concentrations in the leaves and flowers compared to the roots. The levels of phenolics in the roots are probably higher in winter when they overwinter and concentrate in their cells. The lower phenolic content in the roots compared to the flowers and leaves is also due to the extraction method. The size of the extracted plant material also has a certain influence on phenolic extraction. Smaller plant parts are easier to extract with solvents than larger ones [4]. Petals and leaves are thinner, and the extraction solvents come closer to a larger part of the cells, so the transition is easier and faster than in the roots of the purple coneflower, which have a rougher structure.

### 3.2. Comparison of the Phenolic Contents Among Different Extraction Solvents

In this research, pure water (H_2_O), 40% ethanol (40% EtOH), glycerol (Gly), and 5% acetic acid (5% AA) were used for the extraction of phenolics. Figure 1 shows two individual phenolics and four phenolic groups gained with three parts of purple coneflower by four extraction solutions and three various times of extraction. All four extraction solvents certainly affected extraction yield of phenolics (Table 2 and Table 3; Figure 1 and Figure 2). The differences can be attributed to the diverse accessibility of extractible components, which is due to the different chemical structure of the plant [6], the polarity of solvents, and the solubility of the phenolics in these solvents [37].

Among various extraction solvents, Sultana et al. [6] and Butsat and Siriamornpun [38] reported that aqueous ethanol solvent has proven to be an affective solvent to extract phenolic compounds from different plants. The best proved to be a higher percentage of organic solvent (ethanol) and a lower percentage of water [39]. Mokrani et al. [40] reported that no single solvent is capable of efficiently extracting all phenolic compounds. In our study, of all four types of extraction solvents investigated, water was the poorest for extracting the total phenolics in all parts of the purple coneflower, such as the flowers, leaves, and roots (Table 3). In our study, 40% EtOH was the perfect extraction solvent for the efficient extraction of chicoric acid in leaves and roots, which contributed to a high total content of phenolics analyzed (Figure 1; Table 3). Beside chicoric acid, hydroxycinnamic acids were best extracted in water or water with the presence of ethanol (Figure 1). By adding an organic solvent to water, a moderately polar environment is created, which improves the extraction of phenolic compounds. This is in agreement with previous studies [37,41,42].

The flavonols from the flowers and leaves of purple coneflower can also be better extracted in 40% EtOH, mainly because of the higher proportion of kaempferol derivatives, which can be better extracted in the presence of the organic solvent [43]. The flavonols from the roots consist of a higher presence of quercetin derivatives, which can be better extracted in water and accordingly achieve the highest yield in the water solvent [43].

Anthocyanins were detected only in *Echinacea purpurea* flowers and the highest content we obtained with 40% ethanol (Figure 1). Anthocyanin extraction is more efficient when organic solvent also contains a share of water compared to using pure alcohol or water alone [44]. Previous results have shown that ethanol concentration promotes the diffusion of anthocyanins, especially below 60% ethanol [45]. In addition to 40% ethanol and water, we also examined the solvents glycerol and 5% acetic acid, which produced the highest yield of phenolics from the flowers of the purple coneflower. Of the individual phenolics and some phenolic groups, caftaric acid was best extracted in 5% acetic acid and glycerol of all three plant parts examined. Glycerol is harmless, and an affordable component used for the extraction of many medicinal herbs into products for various health disorders [46]. Glycerol, either used alone or in combination with water, has proven to be an effective solvent for extracting polar polyphenolic compounds that exhibit lower solubility in water/ethanol mixtures [47].

Also 5% acetic acid was the best extraction solvent for caftaric acid in all parts of the plant because acetic acid reduced the pH of the solvent. Studies have confirmed that lower pH values (pH 4.00 and 5.00) are best suited for the extraction of phenolics [48]. If we added acid to the solvent, the stability of the phenolics increased [40]. In addition, the pH value is a decisive issue for the enzyme activity expression. Polyphenol oxidases (PPO) are degradative enzymes and are usually active at a neutral pH value. The pH value influences the ionization state of amino acid side chains and/or the ionization of the substrate [49].

Chicoric acid, caftaric acid and other caffeic acid derivatives are highly prone to enzymatic degradation and oxidation in both alcoholic and aqueous solvents [50,51]. Chicoric acid and other phenolic compounds mostly degraded with polyphenol oxidases (PPO) [50]. By adding acetic acid in our study, we were able to increase the extraction efficiency of some phenolics, which is due to a lower pH and inhibition of enzymatic oxidation and maintenance of extraction [52]. In addition, Khoddami and co-authors [5] reported that it is very important to acidify the solvent with acids.

For the development of extracts intended for food or pharmaceutical applications, it is essential to perform additional evaluations, e.g., stability tests, bioactivity assessments, and pH sensitivity analyses. These assessments ensure the quality, efficacy and functionality of the final extract obtained with the selected solvent.

### 3.3. Comparison of the Phenolic Contents Among Different Extraction Time

The amount of phenolics reacts differently to the duration of maceration (Figure 1; Table 3). With longer maceration times, the phenolics in the leaves and roots of the coneflower decrease significantly, while no clear trend can be seen in the flowers (Table 3; Figure 1). Dent et al. [37] found that prolonged time of the extraction potentially decreases the contents of components, particularly due to solvent loss through evaporation. The decrease in phenolics over time can also be explained by Fick’s second law of diffusion, which predicts an equilibrium between the concentration of solutes in the solid matrix and the bulk solution after a certain time [40]. Generally, also in our study, total analyzed phenolics exhibited the highest levels after 3 days of maceration (Table 3; Figure 1 and Figure 2).

Phenolics from flowers and roots diluted in glycerol in the first 6 days responded with a decrease and afterward with an increase in their contents. Also, caftaric and chicoric acid from flowers diluted in 40% EtOH decreased from 3 to 6 days and increased back in day 9 (Figure 1). Apparently, the best time for extracting purple coneflower flowers in ethanol and water solvent was after 6 days of dilution. On the third day, no notable differences were observed between the two solvents (ethanol and water), but after 6 days of dilution, the phenolic content in water was almost twice as high as in 40% EtOH. Adding water to ethanol increased the extraction degree; however, an excessive amount of water in the extraction solution increased the co-extraction of other components, resulting in lower phenolic content in the extracts [53]. After 9 days of extraction, the order was reversed, with higher phenolic yields in 40% EtOH than in water.

In purple coneflower leaves, in which the phenolic content decreased significantly with increasing maceration time, the contents in water solvent had decreased by more than half after 3 to 6 days of maceration and by a further third after day 9. All phenolics in the roots, with the exception of flavonols and flavanones, decreased by more than 70% from the 3rd to the 6th day, and on the 9th day, the phenolics in the roots decreased for an additional 10% of their content. As with the flowers, the phenolics from the roots in glycerol decreased from the 3rd to the 6th day but then increased again to the initial values or even above (total flavanones, hydroxycinnamic acids, flavonols, and TAP) (Table 3). The content of phenolics in 5% acetic acid decreased with the duration of exposure to the solvent and was lower on day 9 than on day 3, with the exception of flavonols in the roots, for which there were no statistical differences between the interaction of solvent and extraction duration (Table 2). Their levels increased at the beginning but decreased thereafter.

The observed reduction in extracted phenolics was attributed to degradation or polymerization reactions that produced new components that interacted differently with the extraction solvent [53]. Higher oxidation occured where open bottles or more air were present. In the study by Bergeron et al. [54], full bottles were prepared to reduce enzyme and oxidation activity. In our study, the bottles were open and, accordingly, many phenolics decreased over time, which was due to the presence of oxygen. Chicoric acid is very sensitive to the presence of atmospheric oxygen [50], which was also evident in our study, where chicoric acid decreased with maceration time among all solvents tested.

To summarize the results of our study, we propose potential practical applications, particularly in the pharmaceutical industry. While shorter maceration times may be economically advantageous, especially at an industrial scale, achieving maximum extraction of bioactive compounds—particularly phenolics—cannot always be without compromising yield or quality. The kinetics of passive diffusion and the interactions between solvent and compound often take time to reach equilibrium, especially in conventional maceration. In addition, intensive research is still underway to develop and optimize methods that could increase extraction efficiency while reducing processing time, such as ultrasound-assisted extraction, microwave-assisted techniques or enzymatic treatments. These approaches aim to maintain or even improve the extraction of valuable phenolic compounds while taking into account industrial scalability and cost efficiency.

## 4. Conclusions

Plants serve as valuable sources of bioactive compounds with health-promoting properties, making them suitable candidates for use as functional foods. Among them, purple coneflower stands out due to its high concentration of phenolic compounds. The results of this study showed that the phenolic yield is significantly influenced by several factors, including the plant part, the solvent for extraction and the maceration time. The leaves and flowers of the purple coneflower yielded the highest values of total phenolics. The roots exhibited the lowest levels of phenolic compounds, probably due to the harvest timing and the larger particle size of the plant material used for extraction. This study gives us an insight into what most influences the yield of phenolics in the various health-promoting extracts from purple coneflower. The structure of the phenolic compound undoubtedly plays the main role, followed by the solvent polarity, its pH, maceration time, the part of the plant used for extraction and the availability of oxygen in the vicinity.

The best product from purple coneflower is extract after 9 days of dilution in glycerol. A high phenolic yield was also obtained with acetic acid after 3 days. Both solvents ensure better solubility of the phenolics from the flowers of the purple coneflower. In summary, the optimal conditions for the extraction of phenolic compounds from the flowers of purple coneflower were the solvent glycerol and an extraction time of 3 days, from the leaves, 40% ethanol, after 3 days of extraction and from the roots, 5% acetic acid, after 6 days of extraction, which yielded the highest amount of phenolics.

Future research should focus on exploring innovative methods of incorporating phenolic substances into dietary supplements, such as encapsulation techniques to improve their stability and enable controlled release. In addition, conducting comprehensive digestibility and bioavailability studies is critical to understanding the gastrointestinal behavior of these compounds and their potential for systemic absorption.

## Figures and Tables

**Table 1:** The list of identified phenolic components by HPLC MS2 in various *Echinacea purpurea* parts.

	*Echinaeca pupurea* Part			Fragmentation
Phenolic Component	Roots	Flowers	Leaves	MS MS^n^
ANTHOCYANINS *				
Cyanidin-3-glucoside		X		449→287
Cyanidin-3-malonylhexoside		X		535→449→287
FLAVONOLS				
Kaempferol hexoside	X			447→285→257,229
Kaempferol-3-glucoside			X	447→285→257,229
Kaempferol-rhamnosyl-hexoside	X	X	X	593→447→285
Kaempferol-acetyl-hexoside		X		489→285
Quercetin-3-galactoside		X		463→301
Quercetin-3-glucoside		X		463→301
Quercetin-3-rutinoside		X	X	609→301
Quercetin-pentoside	X		X	433→301
Quercetin-malonyl-hexoside		X		549→505,463→301
Quercetin-hexoside	X			463→301
Quercetin-3-rhamnoside		X	X	447→301
FLAVANONES				
Naringenin hexoside	X	X	X	433→271→151
HYDROXYCINNAMIC ACIDS				
Chlorogenic acid	X	X		353→191,179,135
Caffeic acid hexoside		X	X	341→179,161→135
Dicaffeoylquinic acid	X	X		515→353→179,173
*p*-coumaric acid hexoside		X	X	325→163,119
*p*-coumaric acid pentoside	X	X	X	295→163,119
Ferulic acid pentoside	X	X	X	325→193→149,134
*trans-*Caftaric acid	X	X	X	311→179,149,135
*cis-*Caftaric acid	X	X	X	311→179,149,135
*trans-*Chicoric acid	X	X	X	473→311→293,179,149,131
*cis-*Chicoric acid	X	X	X	473→311→293,179,149,131

* [M+H]^+^ (*m*/*z*) Anthocyanins were detected in positive ion mode, whereas other phenolics were identified in negative ion mode as [M–H]^−^ (*m*/*z*).

**Table 2:** Statistically significant and not significant differences among different studied treatments for individual, grouped, and total analyzed phenolics.

	Caftaric Acid	Chicoric Acid	Total Hydroxy-Cinnamic Acids	Total Flavonols	Total Flavanones	Total Antho-Cyanins	Total Analyzed Phenolics
Part	***	***	***	***	***		***
Time of maceration	***	*	NS	NS	*	**	*
Solvent	***	***	***	***	**	***	***
Time of extraction x Solvent	*	*	*	NS	*	***	NS
Part X Solvent	***	***	***	***	**		***
Part X Time	NS	NS	NS	NS	***		NS
Part X Time X Solvent	***	***	***	***	***		***

*—significant difference at *p* < 0.05; **—significant difference at *p* < 0.001; ***—significant difference at *p* < 0.0001; NS—no significant difference.

**Table 3:** The content of total analyzed phenolics (mg per 100 g DW) in different parts of purple coneflower (flowers, leaves, roots) under different maceration times and using various extraction solvents.

Time of Maceration	Extraction Solvent	Flowers	S	T x S	Leaves	S	T x S	Roots	S	T x S
3 days	40% EtOH	91.01 ± 10.53	c	E	1022.43 ± 34.38	a	A	1011.32 ± 31.49	a	A
	5% Acetic acid	1696.05 ± 91.47	b	B	845.93 ± 16.15	c	C	493.31 ± 16.73	b	B
	Glycerol	2796.94 ± 93.75	a	A	932.79 ± 13.40	b	B	266.11 ± 11.41	c	DE
	Water	60.13 ± 5.54	c	E	222.59 ± 10.79	d	F	18.74 ± 0.83	d	H
6 days	40% EtOH	98.72 ± 12.01	c	E	483.51 ± 17.66	b	E	251.84 ± 4.77	b	DE
	5% Acetic acid	2104.50 ± 95.83	a	B	809.16 ± 58.80	a	C	330.67 ± 3.75	a	C
	Glycerol	1056.74 ± 92.04	b	E	412.94 ± 13.48	b	E	233.24 ± 9.08	b	E
	Water	169.23 ± 23.13	c	D	66.98 ± 2.94	c	G	142.20 ± 6.29	c	F
9 days	40% EtOH	97.31 ± 4.82	c	E	217.16 ± 8.64	b	F	83.91 ± 1.83	c	G
	5% Acetic acid	1093.74 ± 19.56	b	D	624.45 ± 20.61	a	D	232.4.39 ± 4.39	b	E
	Glycerol	2624.83 ± 91.38	a	A	239.12 ± 9.20	b	F	289.19 ± 9.38	a	D
	Water	39.57 ± 2.59	c	E	39.87 ± 1.94	c	G	39.60 ± 2.15	d	H

Different letters (a–d) in the columns indicate statistically significant differences in TAP content of purple coneflower flowers, leaves, and roots between various solvents for the extraction (S) (separately for each extraction time). In addition, different capital letters (A–H) in the columns represent significant differences between the two analyzed factors (T × S—time of maceration and solvent) with the LSD range test (*p* < 0.05).

## Data Availability

The original contributions presented in this study are included in the article/Supplementary Materials. Further inquiries can be directed to the corresponding author.

## Supplementary Materials

The following supporting information can be downloaded at: https://www.mdpi.com/article/10.3390/foods14132325/s1, Figure S1: The content of total analyzed phenolics among different treatments for purple coneflower flowers in mg per 100 g DW; 5% AA—5% acetic acid, EtOH—ethanol, Gly—glycerol, H_2_O—water.
