# Supplementary figures and images for: Innovative Approaches to Extracting Phenolics from Echinacea purpurea: Maximizing Yield and Efficacy
Source: Foods. 2025 Jun 30;14(13):2325. doi: 10.3390/foods14132325 (PMC12249240; doi:10.3390/foods14132325)

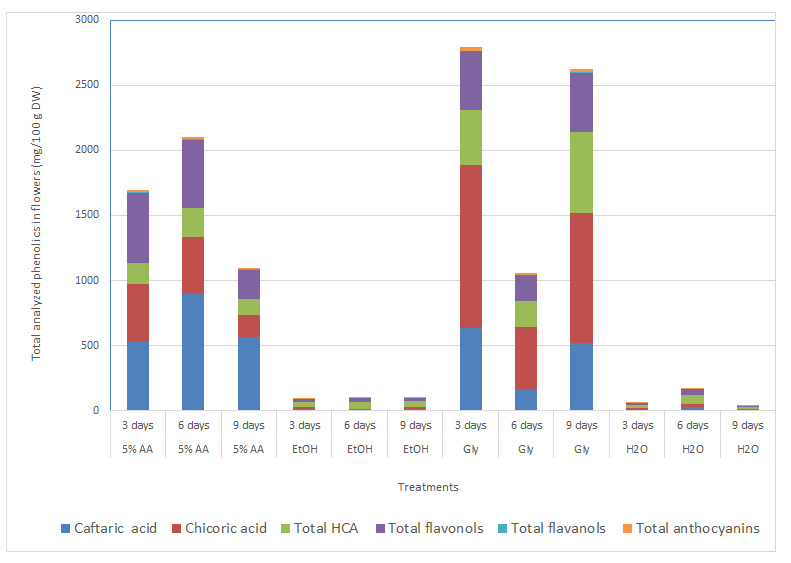

Supplement: Supplementary file 1 [file foods-14-02325-s001.zip › foods-3698557-supplementary.png]
